# Seroprevalence and Risk Factors Investigations of Parvovirus Disease in Tibetan Pigs: First Report from Tibet

**DOI:** 10.3390/vetsci9100576

**Published:** 2022-10-17

**Authors:** Feifei Yan, Shijun Xu, Zhenyu Chang, Mudassar Nazar, Yangzom Chamba, Peng Shang

**Affiliations:** 1 College of Animal Science, Tibet Agriculture and Animal Husbandry College, Linzhi 860000, China; 2 The Provincial and Ministerial Co-founded Collaborative Innovation Center for R & D in Tibet Characteristic Agricultural and Animal Husbandry Resources, Linzhi 860000, China; 3 University of Agriculture Faisalabad, Burewala 61010, Pakistan

**Keywords:** porcine parvovirus, risk factors, seroprevalence, Tibetan pig

## Abstract

**Simple Summary:**

Porcine parvovirus disease is an animal disease with a high infection rate that is wide spread in the world. Its spread and variants have a very serious impact on large-scale livestock economy. So far, there have been no reports on the infection and genetics of pig parvovirus in Tibetan pigs. This study was conducted to investigate and analyze the seroprevalence, regional distribution and risk factors responsible for porcine parvovirus in Tibetan pigs in Tibet. This study also contains an effective response and analysis of strategies made in the areas under investigation through the epidemic, distribution and risk factors inquiry of the virus. A total of 356 serum samples were collected from four counties in Tibet, and the anti-porcine parvovirus antibodies were detected by using the competitive ELISA kit. In this investigation, 324 (91.01%) of 356 serum samples were found to be positive for porcine parvovirus. Porcine parvovirus is also extremely widespread in Tibet, reaching a worrying seroprevalence that requires greater awareness of this disease by the operators in the swine sector. Possible measures to improve surveillance and monitoring of the disease should be considered.

**Abstract:**

Porcine parvovirus (PPV) disease is a worldwide spread animal disease with high infection rate and serious impact on meat economy causing significant losses in livestock production. The purpose of this paper is to investigate and analyze the regional seroprevalence of PPV in Tibetan pigs in Tibet and evaluate risk factors related to the disease. A total of 356 serum samples of Tibetan pigs were collected from four counties and districts in Tibet, and anti-PPV antibodies were detected by using a commercial competitive ELISA. Our results show a seroprevalence of 91.01% (324 serum samples were found to be positive for anti-PPV antibodies). The positive rate among different district was 100%, 96.55%, 93.68% and 72.83%, respectively in the Mainling County, in Bayi district, Nang County and Bomê County. We found significant differences between different age and gender groups; particularly female animals show a seroprevalence of 96.03% while the males only 83.46%. From the perspective of the growth stage, our results indicate that subadults show a seroprevalence significative higher than other age groups (100%). This study describes for the first time the PPV seroprevalence among Tibetan pigs characterizing risk factors involved in its transmission and providing information to be taken into account for eventual surveillance or eradication plans.

## 1. Introduction

PPV disease is an infectious disease caused by porcine parvovirus, a single-stranded and non-enveloped DNA virus first discovered in 1965 in Germany [1]. This virus shows a small genome and belong to the genus Parvovirus, subfamily Parvovirinae, and family Parvoviridae (https://talk.ictvonline.org/taxonomy/, accessed on 1 August 2022) [2]. PPV results in huge losses in the pig industry over the whole world thanks to its widespread and relevant resistance in the environment [3,4,5,6,7,8,9]. The infection primarily occurred during an oro-fecal cycle and resulted in abortion [10,11,12], stillbirth, and fetus mummification in gestating sows, as well as diarrhea, skin injury, myocarditis, and high mortality in suckling piglets [13,14,15,16], which explains its impact on the swine industry, particularly in the absence of vaccination plans. Coinfections with other viruses (PCV, PRRSV, PEDV, etc.) are very common and exacerbate the health conditions of the piglets [17]. The spread among different herds usually occurs with feeding, pollutants, and inanimate carriers (as shoes, clothes, and car wheels) and sometimes results in large-scale and cross-regional transmission [2].

Different studies have revealed that PPV has a wide geographical distribution and has been detected in many parts of the world, often without any measures being carried out to stop the spread of the infection. In addition, studies have shown that PPV has genetic diversity [18], as in the past years many other PPV genomes (PPVs) have been found in pigs and continuously identified by high-throughput sequencing and named PPV2- 7 infections under different names [3,19,20,21,22,23]. China is also conducting epizootiological investigations, including the isolation and identification of PPV strains in many places [17]. PPV3, PPV4 and PPV7 were found in the UK, Brazil and South Korea, respectively [8,24,25]. PPV2, PPV3, and PPV4 were detected in Romania, Croatia, Thailand, Japan, and South Africa [26,27,28,29,30]. All of these PPV species were detected in China, the United States, and Poland [27]. The first isolation in China dates back to 1983 [31]. After that, the virus was noticed to be widespread among pigs in China, including new strains [32,33,34,35], posing a serious threat to China’s pig industry. Local and small-sized herds (which have bred local pig breeds such as the Tibetan pig) are the weakest entities in this scenario and require special attention when it comes to infectious diseases. Tibetan pigs are mainly distributed in the Qinghai-Tibet Plateau, with an average altitude of more than 4000 m. They show significant phenotypic and physiological differences from lowland pig breeds as they are rare plateau pig breeds [36]. It is primarily found in China’s Qinghai-Tibet Plateau. Tibetan pigs have the characteristics of adapting to harsh climates. Due to living in high altitude and low air pressure areas for a long time, they have adapted to the environment of hypoxia and low temperature [37], and the research shows that Tibetan pigs originated from the Qinghai-Tibet Plateau and survived in the wild. It is one of the main food and economic sources for Tibetan nomads in China [38]. However, its low fecundity makes it a valuable local variety resource in China. In addition, due to the environmental constraints in Tibet, a lack of awareness in farmers and the lack of surveillance have greatly increased the risk of transmission of PPV, which may lead to serious economic losses. So far, there have been no reports on the infection and genetics of pig parvovirus in Tibetan pigs, and this study aims to investigate and analyze the epizootiological situation of PPV disease (PPVs) in Tibetan pigs, as well as provide a reference for future PPV research.

## 2. Materials and Methods

### 2.1. Study Site

**Figure 1:**
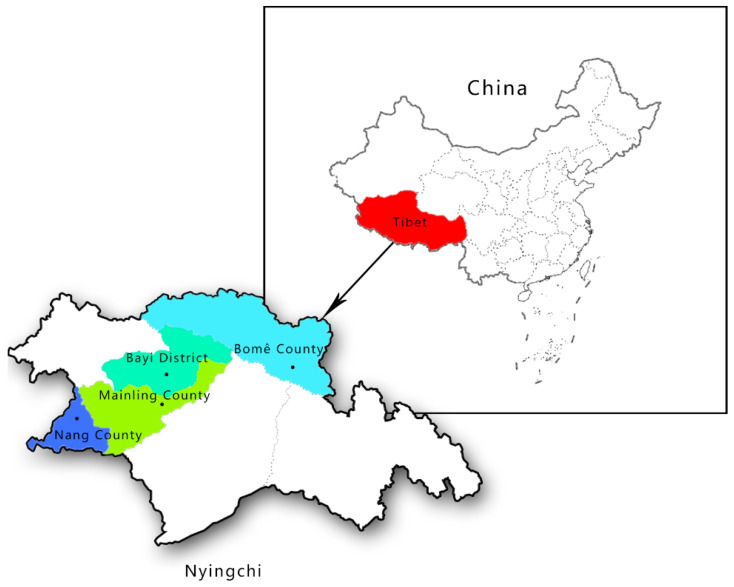
Map of the sampling area: Qinghai Tibet Plateau (with an average altitude of more than 4000 m), Nyingchi City (with an average altitude of about 3000 m).

This study site is located in the southeast of Tibet. Informed consent for the use of Tibetan pig blood was obtained from all subjects involved in the study. By random sampling, a total of 356 samples of different gender, age and health conditions were collected from four different counties and districts in Nyingchi City, Tibet under the approval of national animal welfare (SKLAB-2012-04-07) (Figure 1).

### 2.2. Samples Collection and Processing

In this study, all the samples were collected from the anterior vena cava of pigs. The blood was collected, and serum was prepared according to the conventional method. The serum was clear without hemolysis and pollution. All serum samples were tested for antibodies to PPV using a commercial enzyme linked immunosorbent assay (ELISA, Beijing Yisen Biotechnology Co., Ltd., Beijing, China) kit was used to detect IgG antibody levels in PPV according to the kit instructions. The sample OD value ≥ 0.8 was positive and the sample OD value < 0.8 was considered as negative.

### 2.3. Statistical Analysis

The data were calculated by chi-square test using SPSS 26 software with a statistically significant level when the probability *p* value was *p* < 0.05, and the 95% confidence interval of the overall rate was estimated using the normal approximation method and the exact probability method, and the relative risk of the identified factors was calculated by the ratio between the two groups.

## 3. Results

### 3.1. Seroprevalence of PPV

Among the 356 samples tested, in 324 samples, antibodies were detected against PPV, obtaining a seroprevalence of 91.01% (Table 1). All the sampling units were positive. We found different seroprevalence among different sampling districts: 100% in Mainling County, 96.55% in Bayi District, 93.68% in Nang County, and 72.83% in Bomê County. Considering different age groups, the positive rate is 100.00% in the sub-adults, 92.06% in the juveniles, and 83.46% in the adults. We found a higher seroprevalence in females (96.03%) than in males (87.32%). Considering the different health conditions of pigs, the positive rate of pigs showing clinical symptoms attributable to PPV is 100.00%, while that of apparently healthy pigs is 90.80%. We evaluated three possible risk factors, including location, age and sex, through our data and showed that these three influencing factors are associated with an increased risk of PPV sero-positivity in Tibetan pigs.

None of the Tibetan pigs we collected blood had been vaccinated against PPV, and we also checked the PPV positive rate of unvaccinated animals in other parts of the world. The results are shown in Table 1. As can be seen from the table, compared with other regions, the positive rate of PPV virus in Tibet, China is much higher than that of other unvaccinated regions in the world.

### 3.2. Epizootiological Calculation

In this study, a total of 356 samples of different gender, age and health conditions were collected from four different counties and districts in Nyingchi City, Tibet. The positive rate of the serum of Tibetan Pig parvovirus was calculated, the data were tested by the program in SPSS 26 software with 95% confidence interval and the odds ratio of risk factors were calculated. The results of the calculation for the epizootiological odds ratio of different locations, age and gender in Table 1 and Table 2 show that, Bayi District, the adults and the high relative risk of being female during production are the linked risk factors.

## 4. Discussion

Epizootic prevention of infectious diseases has always been an important point issue in the agriculture industry. PPV [2] is a widespread virus which has been detected in many places in the world [18], including China. In past years, many other PPV genomes have been found in pigs and continuously identified by high-throughput sequencing and named PPV2-7 infections under different names [3,19,20,21,22,23], while all these PPV species were detected in China. In the reports of relevant PPV diseases in China, the infection rate is about 60% [35].

Although there is a vaccination plan for the pigs with large-scale breeding in China, the awareness concerning this disease in local and small size farms is very low. Moreover, due to its vast territory, geographical isolation among counties, underdevelopment the epizootiological status of this herds is unknown. Tibet is mainly distributed in Nyingchi, Shannan and Qamdo and the Tibetan pig is a small local pig breed in this area. In previous investigations there are no reports on pig breeds in Tibet related to this infection. Therefore, this study is also the first investigation and study on the infection of Tibetan pigs with PPV in the Tibet region of China.

This study found that in the Tibetan pig population being investigated, the positive rate of PPV was generally very high, with an average infection rate of 91.01%. The highest positive rate has been found in the areas closely connected with the main urban area in Nyingchi city. This result may be explained by the smaller distance to intensive farms than other district object of the sampling as well as to a higher number of inanimate and animate vectors (people, transport, etc.).

The seroprevalence value found around the world varies greatly. An extensive study conducted in Asia (India) discovered that 1.24% of feral pigs are bred in the northeastern regions, while 39.1% are bred in Punjab state [39]. In Uganda, the seroprevalence varies depending on the district studied. In fact, seroprevalence rates of 6.2% and 3.4% have been reported in the Masaka and Lira districts, respectively [40]. This percentage increased when seroprevalences were observed in sows with PPV clinical outcomes, as revealed in a Thai study that discovered antibodies against PPV in 86% of the sampling animals [41]. In Trinidad and Tobago, a seropositive rate of 28% was recently discovered [42].

Given the spread of the infection, more emphasis must be placed on wild animals (wild boars), which frequently serve as a reservoir for multiple infections [43]. Thus, it appears that PPV disease in wild swine is a source of infection for domestic pigs. In this case, too, the outcomes are highly variable. Seroprevalence was found to be 78% in Sweden, but only 2.2% in Turkey [44,45]. Furthermore, as is often the case in serological investigations, the outcome is determined not only by the epizootiological situation but also by the performance of the test used, the characteristics and dimensions of the sampling population, and other variables [46].

We discovered that antibody prevalence in wild swine is correlated with age, with seroprevalence higher in subadults after testing pigs at various feeding and age stages. This is an unexpected outcome. In other studies, for several infectious diseases, higher seroprevalence is frequently found in juveniles, who are more susceptible to these infections [47]. We also took into account that subadults and adults had more time to come into contact with the etiological agent and develop antibodies. We can understand this data because the animals on this farm were bred without any division into classes and without physical barriers, so all age classes were in contact with each other, which increased the spread of PPV.

We also discovered that females have significantly higher seroprevalence than males. Several surveys have found that female wild boars and domestic swine are more susceptible to infectious diseases than males [48]. We can understand this result by considering the spreads within and between maternal groups: the sow’s proximity to piglets (the main spreader and eliminator of PPV) and other sows (elimination via aborted fetus and placentas). Contact between adult females and young animals is more likely than contact between adult males and young animals. Furthermore, Tibetan pigs are typically bred in large systems where the animals live in gregarious groups consisting of a dominant matriarch, sows, and mothers with piglets. As expected, we found a higher seroprevalence in those showing diarrhea symptoms, who obviously have a higher chance of being in contact with this virus and being seropositive. This work provides new insight regarding the spread of PPV in China. Further research is necessary to assess the pathogen’s role in the Tibetan pig population, including molecular approaches to determine the circulating genotypes and fully understand the dimension of the problem in China.

## 5. Conclusions

Our findings confirm that PPV is endemic in Tibet’s swine population, which is consistent with what has been reported in other Chinese regions and countries. According to current research, the PPV infection rate in Tibet is extremely high. Continuous surveillance is strongly advised in order to describe the epizootiological situation, monitor infection prevalence, and propose containment measures. Some variables, such as age, gender, clinical symptoms, and district, have been linked to PPV exposure. Tibet, the study’s chosen location, has distinct geographical characteristics and environmental diversity. Our findings highlight the importance of continuing this type of survey in the Tibetan region and throughout China in order to preserve this diversity as well as the rural economy and the development of this area.

## Figures and Tables

**Table 1:** Relationship between Tibetan pig characteristics and PPV serological status, and corresponding 95% confidence interval (CI), *p* value.

Factor	*n*	Positive	%	95%CI	*p*
Total	356	324	91.01%	87.5–93.8	
Gender					
Male	205	179	87.32%	82.0–91.5	*p* = 0.005
Female	151	145	96.03%	91.6–98.5	
Age					
Juveniles	133	111	83.46%	76.0–89.3	*p* = 0.014
Sub-adults	97	97	100.00%	96.3–100.0	
Adults	126	116	92.06%	85.9–96.1	
Health status					
Diseased	8	8	100.00%	63.1–100.0	*p* = 0.369
Healthy	348	316	90.80%	87.3–93.6	
District					
Bayi district	29	28	96.55%	82.2–99.9	*p* = 0.237
Mainling County	140	140	100.00%	97.4–100.0	
Bomê County	92	67	72.83%	62.6–81.6	
Nang County	95	89	93.68%	86.8–97.6	

**Table 2:** Relative risk relationship among different characteristics of Tibetan Pig parvovirus.

			Exposure Factors						
			Sites			Age		Gender	
			Bayi	Bomê	Nang	Juveniles	Adults	Male	Female
Non exposure factors	Sites	Bayi District	1	0.10	0.53				
		Bomê County	10.45	1	5.53				
		Nang County	1.89	0.18	1				
	Age	Juveniles				1	2.30		
		Adults				0.43	1		
	Gender	Male						1	3.51
		Female						0.28	1

## Data Availability

The data presented in this study are available on request from the corresponding author.
